# Pharmacogenomics of amlodipine and hydrochlorothiazide therapy and the quest for improved control of hypertension: a mini review

**DOI:** 10.1007/s10741-018-09765-y

**Published:** 2019-01-15

**Authors:** Rabia Johnson, Phiwayinkosi Dludla, Sihle Mabhida, Mongi Benjeddou, Johan Louw, Faghri February

**Affiliations:** 1 Biomedical Research and Innovation Platform (BRIP), South African Medical Research Council (SAMRC), Tygerberg, 7505 South Africa; 2 Division of Medical Physiology, Faculty of Medicine and Health Sciences, Stellenbosch University, Tygerberg, 7505 South Africa; 3 Department of Biotechnology, Faculty of Natural Science, University of the Western Cape, Private Bag X17, Bellville, Cape Town, 7535 South Africa; 4 Department of Haematology, Faculty of Medicine and Health Sciences, Stellenbosch University, Tygerberg, 7505 South Africa

**Keywords:** Amlodipine, Blood pressure, hydrochlorothiazide, Hypertension, Single nucleotide polymorphisms (SNPs)

## Abstract

Blood pressure (BP) is a complex trait that is regulated by multiple physiological pathways and include but is not limited to extracellular fluid volume homeostasis, cardiac contractility, and vascular tone through renal, neural, or endocrine systems. Uncontrolled hypertension (HTN) has been associated with an increased mortality risk. Therefore, understanding the genetics that underpins and influence BP regulation will have a major impact on public health. Moreover, uncontrolled HTN has been linked to inter-individual variation in the drugs’ response and this has been associated with an individual’s genetics architecture. However, the identification of candidate genes that underpin the genetic basis of HTN remains a major challenge. To date, few variants associated with inter-individual BP regulation have been identified and replicated. Research in this field has accelerated over the past 5 years as a direct result of on-going genome-wide association studies (GWAS) and the progress in the identification of rare gene variants and mutations, epigenetic markers, and the regulatory pathways involved in the pathophysiology of BP. In this review we describe and enhance our current understanding of how genetic variants account for the observed variability in BP response in patients on first-line antihypertensive drugs, amlodipine and hydrochlorothiazide.

## Introduction

Hypertension (HTN) often called the “silent killer” is defined as a recurrent systolic/diastolic Blood  pressure (BP) higher than 140/90 mmHg. It affects approximately 1.13 billion people with an estimated 7.5 million deaths globally [1]. Of great concern is the 30–40% of working age adults (25–64 years of age) that are hypertensive, and of which only one third have their BP under control [1, 2]. Uncontrolled BP is associated with increased risk for stroke and coronary artery disease, which are the leading causes of death worldwide. Hypertension has not only become a public health problem but a key national and international priority as uncontrolled HTN imposes a heavy cost on a country’s national budget, health care services, and individual households. Similarly, in Sub-Saharan Africa, HTN is classified as the number one mortality risk with an estimated 500,000 deaths, affecting approximately 10 million South Africans with an associated 60–75% uncontrolled cases [3, 4]. Furthermore, only 27% of HTN patients are aware of their status, and only 18% are on treatment [5]. More alarmingly, HTN is a multifactorial disease that occurs in clusters with other diseases such as cardiovascular diseases and type 2 diabetes mellitus (T2DM) [4]. This is of great concern, as the latter two diseases contribute to the exacerbating non-communicable disease burden.

## Hypertension management and clinical practice guidelines

Current interventions in the management and control of HTN aim to reduce the mean BP of an individual, with the goal of achieving consistent BP control. Thus, apart from diet and exercise, control of HTN can be achieved through patients’ adherence to the prescribed anti-HTN drug therapy. Anti-HTN therapy includes the use of angiotensin-converting enzymes inhibitors (ACEIs), angiotensin receptor blockers (ARBs), calcium channel blockers (CCBs), and thiazide diuretics [6, 7]. These prescribed drugs can be administered as a monotherapy, a two-drug fixed-dose combination, or three-drug combination pill with the aim to increase treatment adherence [2, 8–10]. However, according to the American Hypertension Association, prescribed anti-HTN drugs should be administered in the following combinations that are based on a drug’s efficacy, safety, and tolerability: (i) ACEI/CCB, (ii) ACE/thiazide diuretic, (ii) ARB/CCB, and (vi) ARB/thiazide-type diuretic [2]. Whereas, the combination of ACE with ARB is restricted to use within the same regimen. The latter was confirmed in an evidence-based recommendation review performed in 2014 by medical consultants. The authors stated that an initial anti-HTN monotherapy should include any of the prescribed anti-HTN drugs, ACEIs, CCBs, ARBs, or thiazide diuretics but in a two-drug therapy, while ACEIs and ARBs should not be administered together [7]. They further stated that CCBs and thiazide diuretics should be used as first-line therapies, particularly in the Black population, as it has been associated with an improved BP control in the African-American population [7]. The panel continued stating that if BP is not attained within the first month the recommended drug dose should be increased. If neither monotherapy nor combination therapy works, a third drug should be added, and the best triple fixed-dose combination include amlodipine from the CCBs, valsartan from the ARBs, and hydrochlorothiazide (HCTZ) from the thiazide diuretics [11]. To confirm this, various cross-sectional studies on the different classes of anti-HTN drugs and their effectiveness to control BP have been investigated [12–14]. In a study done in 954 hypertensive individuals and patients with T2DM, the authors confirmed that the use of CCBs and thiazide diuretics was superior to any other anti-HTN drug class [13]. A similar observation was made in a longitudinal study conducted between January 2005 and April 2011 on 2780 uncontrolled HTN patients. The authors found that different anti-HTN drug classes displayed different effects on BP control, and that patients on CCBs and thiazide diuretics showed a greater effect in lowering BP variability [12]. In addition, as CCBs and thiazides are the treatment of choice in the African-American population, it has been proposed that treatment therapy should be initiated with a low dose of thiazide monotherapy and if BP is not achieved a CCB should be added. This has been confirmed in the Jackson Heart Study where it was found that although monotherapy with CCBs in the African-American population is more effective, thiazide diuretics were more effective to obtain and sustain target BP [15]. Needless to say, therapeutic interventions that investigated the effects of mono or combination therapy on the control of BP showed that a low-dose complementary treatment is more effective in reducing BP with a response rate of 75–95% compared to monotherapy. However, although effective, the efficacy of anti-HTN mono or combination therapy is affected by race, gender, age, and inter-individual variation, explaining the observed 60% of HTN patients whose BP remain suboptimal and uncontrolled even after rigorous treatment adherence [4, 14–18]. Thus, this review will elaborate on single nucleotide polymorphisms (SNPs) within drug metabolizing and transporter genes that may contribute to the etiology of inter-individual differences in BP response to two first-line HTN drugs, amlodipine and HCTZ. Therefore, a systematic search on the association between single nucleotide polymorphism, HTN, inter-individual variation, as well as amlodipine and HCTZ was done by probing major search engines and databases such as PubMed/Medline, EMBASE, Cochrane Library Databases, Google Scholar and data submitted to PharmGKB. The search was done from inception until end of June 2018. The search included gray literature such as abstract proceedings and pre-prints, while no language restrictions were implemented, with review articles screened for primary findings. Medical subject heading (MeSH) terms such as hypertension, blood pressure, single nucleotide polymorphism, inter-individual variation, amlodipine, and hydrochlorothiazide, including corresponding synonyms and associated terms for each item, were used.

## Pharmacokinetics and mode of action of first-line hypertensive drugs

### Amlodipine

Amlodipine was introduced more than 35 years ago as a cardiovascular drug [19]. This first-line long-acting 1-4-dihydropyridine (DHP) CCB is known to control BP pressure for 24 h, with minimum side effects. Amlodipine has been shown to act by inhibiting the influx of calcium through L-type calcium channels into vascular smooth muscle cells, preventing vasoconstriction while simultaneously improving blood flow. Amlodipine is prescribed at a daily dose of 5 and 10 mg, whereas the most commonly used derivative, amlodipine besylate, is given at 6.9 mg and may reach bioavailability of 60–90% [19–21]. There are relatively small fluctuations in plasma concentrations across a dosage interval and the prescribed dose is effective for children aged 6 years, whereas a starting dose of 2.5 mg is recommended for the elderly [6, 19]. The pharmacokinetics (ADME; absorption, distribution, metabolism, and elimination) of amlodipine have been reviewed extensively, while its oral administration show a linear dose-related pharmacokinetic profile (Table 1) [26]. Amlodipine is known to be highly bioavailable with a peak plasma concentration at 6–12 h, reaching a steady state 7–8 days after administration of the 5 mg/daily oral dose, with low fluctuation and plasma concentration [2, 19, 22, 23]. Ninety percent of amlodipine is known to be bound to the plasma membrane and the volume of distribution of amlodipine is large (21 L/kg) [27]. Amlodipine is largely metabolized in the liver via the Cytochrome P450 3A4/5 (*CYP3A4/5*) family of enzymes to its inactive form pyridine metabolites (M9), after which M9 will undergo further oxidative deamination to M10, O-demethylation to M1, and O-dealkylation to M4 [22]. Once metabolized, the drug is slowly eliminated for 40–60 h, with 10% of the parent compound and 60% of the metabolites excreted in the urine while fecal excretion account for 20–25% of the drug removal [19].

**Table 1:** Pharmacokinetic properties of amlodipine

Pharmacokinetics	Effect	Reference
Bioavailability	Amlodipine is orally administrated with a bioavailability of 64–90%.	[2, 19, 22]
Absorption	Amlodipine reach peak plasma concentration (t_max_) 6–12 h after administration, while steady state plasma concentrations will be reached within 7–8 days of daily dosing.	[2, 23]
Distribution	Amlodipine has a high volume of distribution (21 L/kg) and a large proportion of the dose is distributed in the tissue with ~ 90% of the circulating drug being bound to the plasma membrane.	[2, 22, 23]
Metabolism	Amlodipine is extensively metabolized in the liver into its inactive metabolites via *CYP3A4/5*.	[2, 22, 23]
Elimination	Amlodipine is slowly cleared with an elimination half-life of 40 to 60 h. If discontinued, BP returned to baseline after 1 week. Urine is the major route of elimination. Amlodipine is converted to inactive metabolites (60%), which are excreted into the urine while 10% of the excreted drug remains unchanged. Amlodipine is converted from dihydropyridinebmoiety to a pyridine derivative (M9). Fecal excretion accounts for 20–25% of the dose	[2, 19, 22, 24, 25]

### Hydrochlorothiazide

Thiazide and thiazide-like diuretics are one of the leading drugs currently used for their antihypertensive properties. Although HCTZ remains a drug of choice for HTN [28, 29], its effective use as a monotherapy can be limited by various factors, mainly those associated with inter-individual variation [30, 31]. The limitations responsible for the reduced efficacy of HCTZ contribute to the rapid rise in mortality in patients with uncontrolled HTN [31]. Other main concerns relating to the use of thiazides are usually their tendency to cause hypokalemia, impair glucose tolerance, and abnormally elevate serum cholesterol and uric acid levels [32]. HCTZ is orally administered with the daily dosage ranging from 25 to 75 mg/day, and an estimated bioavailability of 60–80% [33, 34] (Table 2). It has been established that the gastrointestinal absorption of HCTZ is rapid and peak plasma concentration is achieved at ~ 2 h, and its absorption is enhanced when it is administered with food, with the majority of the drug absorbed in the duodenum and the upper jejunum [34, 35]. HCTZ is transported ~ 40% bound to plasma proteins and it can accumulate in erythrocytes. The ratio between the drug in erythrocytes and plasma averages 3.5 and it has been reported that the concentration of the drug in erythrocytes can be as high as nine-fold the concentration in the plasma [37]. Limited evidence exists concerning the metabolism of HCTZ; however, 2-amino-4-chloro-1,3-benzenedisulfonamide and chlorothiazide are some of the metabolites that have been detected in urine after administration of this drug [36, 38, 39]. The half-life of HCTZ is estimated to be 5 to 14 h, while the main excretion route for this drug is through the kidneys [36]. In addition, the mean renal clearance is ~ 300 mL/min and greater than 95% of the absorbed drug has been shown to be excreted unchanged in urine (Table 2) [36].

**Table 2:** Pharmacokinetic properties of hydrochlorothiazide

Pharmacokinetics	Effect	References
Bioavailability	Oral administration of the drug ranges from 25 to 75 mg/day, and has a bioavailability of 60–80%.	[34, 35]
Absorption	Gastrointestinal absorption of the drug is rapid and peak plasma concentration is achieved at ~ 2 h. With the majority of the drug is absorbed in the duodenum and the upper jejunum.	[34, 36]
Distribution	The drug is transported ~ 40% bound to plasma proteins and it can also accumulate in erythrocytes. The ratio between the drug in erythrocytes and plasma averages 3.5. Another report showed the concentration of the drug in erythrocytes could be as high nine-fold to that of plasma.	[36, 37]
Metabolism	Limited evidence exists related to the metabolism of this drug. However, 2-amino-4-chloro-1,3-benzenedisulfonamide and chlorothiazide are some of the metabolites that have been detected in urine after administration of this drug.	[36–39]
Elimination	The half-life of HCTZ is estimated to be 5 to 14 h. The main excretion route for this drug is the kidneys, with the mean renal clearance of ~ 300 mL/min and greater than 95% of the absorbed drug shown to be excreted unchanged in urine.	[33, 36]

## Pharmacogenomics and inter-individual variation of hypertensive drug therapy, amlodipine and HCTZ

Variations in the human genome due to SNPs are believed to be the most important cause of the variability in an individual’s drug response within a population. Genetic variation frequencies differ between ethnic groups and could lead to susceptibility to adverse drug reactions. This fact that drug response is not universal is one of the main motivations behind personalized medicine. The occurrence of adverse drug reactions (ADRs) due to genetic differences in population is a common phenomenon and is a huge public health concern as it can exacerbate the conditions of the individual patient and contribute to a strain on the health systems in terms of its economic implications. In the USA, alone the cost of ADR-related hospitalizations amounted to $136 billion in the last few years (https://www.hcup-us.ahrq.gov/reports/statbriefs/sb234-Adverse-Drug-Events.pdf-accessed 19 September 2018) of which antihypertensive contribute to 7.9%.

There are various other factors, which contribute to the occurrence of ADRs and variation in drug responses in different individuals. These include age, gender, other underlying disease complications, nutrition, and co-medications to name a few. Prescribing drugs with a potential co-interactions may result in an ADR. However, when these factors are removed, a substantial proportion of ADRs remain present due to a genetic predisposition. The differences in ADR susceptibility in ethnic populations have a big impact on the development of drugs for any disease, where performing a clinical trial in one population, that does not represent the complete target market, might not produce the expected outcome. This is especially of concern when most of the clinical trials are still performed in the developed countries. Therefore, a population-based pharmacogenomics approach is promising to address the issue of ADRs in most settings by integrating the use of functional SNPs and population-differentiated SNPs in drug development and treatment. However, there are limitations to this approach due to complexities that arises because researchers will have to find the SNPs that are not only differently distributed among populations, but they also need to show functional significance. In spite of this, the approach remains valuable in addressing the issue of HTN in the different populations, as it can still identify vulnerable individuals by using identified SNPs linked to ADRs.

Numerous studies have reported on the genetic associations of uncontrolled HTN and the effects thereof on treatment outcome (Tables 3 and 4). These observations have not only enhanced our understanding of HTN and elaborated on mechanisms by which amlodipine and HCTZ produce efficacy, but have also contributed towards unraveling SNPs that may account for the observed differences in BP response to antihypertensive agents. Furthermore, it is known that variation in genes that code for drug metabolizing enzymes as well as drug transporters plays an important role in the regulation and control of HTN. Therefore, results from various genome-wide association studies (GWAS) studies concerning the effect of genetic loci on the responsiveness of hypertensive patients to amlodipine and HCTZ have been summarized in Tables 3 and 4.

**Table 3:** Single nucleotide polymorphisms associated with BP response in genome-wide association analyses of amlodipine-treated patients

Gene	SNP	Treatment outcome	Population	References
*ABCB1*	rs1045642	Gender differences and SNPs in *ABCB1* gene (2677G/T and 3435C/T) showed increased oral clearance of amlodipine. Males require higher concentration of amlodipine.	Asian	[40, 41]
*ACE*	rs200148 rs4291	For SNP rs200148 in the African-American cohort, the A allele frequency is higher in the chlorthalidone treatment group as compared to the amlodipine group. Promoter mutations in rs4291 were associated with lower fasting glucose for the model AA/AT compared to the TT.	African-American and Caucasian	[42]
*CACNA1D*	rs312481 rs3774426	A significant reduction in BP was observed for the combined presence of the identified SNPs (rs312481G/A and rs3774426C/T). Individuals with CC genotype responded better as measured by lowered SBP.	Asian	[43, 44]
*CACNA1C*	rs527974 rs2239050 and rs2238032	Identified SNP 527974G/A was able to decrease BP significantly after treatment. However, none of the identified SNPs were similar to those identified in the Brenner study.	Asian	[43–45]
		Estonian Genome Project found that patients with the combined identified SNP had an increased efficacy to amlodipine treatment outcome.	Caucasian	
*CYP3A4*	rs2246709 rs2740574	Blood pressure response was gender specific and associated with African-American population with genotypes (16090T/C and 392 A/G).	African-American and	[22, 42, 46–48]
		Gender differences was observed but not significant and, CYP3A5*3 does not affect amlodipine efficacy.	Asian	
*CYP3A5*	rs776746	*CYP3A5* 6986A/G was not associated with decrease in BP response in African-American population. But, a significant reduction in BP occurred in Chinese and Korean cohort, CYP3A5*3/*3 carriers exhibited lower plasma amlodipine concentrations than CYP3A5*1 carriers. Gender differences was observed but not significant and, CYP3A5*3 does not affect amlodipine efficacy.	African-American/Asian	[46–49]
*GNB3*	rs5443	Splice variant is associated with the DBP lowering effect of telmisartan but not amlodipine in Chinese.	Asian	[50]
*MDR1*	G2677T/C3435T	Discordant results with the C3435T found that the plasma drug concentration of amlodipine in healthy volunteers of the *MDR1* C3435T mutant allele carrier was lower than that of the CC type. However, according to the study of Cai and colleagues, the MDR1 C3435T mutant did not influence the effect of amlodipine in renal transplant patients with hypertension. Therefore, further investigations are required to elucidate the impact of the *MDR1* C3435T polymorphism on the pharmacokinetics and efficacy of amlodipine.	Caucasian and Asian	[42, 51]
*NOS1AP*	rs10494366	Significance (SNPs at this gene as relevant to stroke pharmacogenetics.	African-American	[52]
*NPPA*	rs5065	The 2238T/C variant had lower event rates were for the C allele carriers than for the TT homozygous when comparing chlorthalidone and amlodipine. The AA genotype responds better to amlodipine.	Multiple races and ethnic groups	[53]
*NUMA1*	rs10898815	Increased response as determined by a greater decrease in DBP but SBP blood pressure.	Multiple races and ethnic groups	[44]
*PICALM*	rs588076	Patients with the GG genotype and hypertension may have a greater decrease in blood pressure.	Multiple races and ethnic groups	[44]
*POR*	rs1057868	1509 C/T genotypes had no significant impact on the blood drug concentration and efficacy of amlodipine due to sample size.	Asian	[42]
*RYR3*	rs877087	Reduced BP response was observed.	Caucasian	[54]
*TANC2*	rs2429427	Reduced BP response was observed.	Multiple races	[44]

*ABCB1* ATP-binding cassette subfamily B member 1, *ACE* angiotensin I converting enzyme, *AGT* angiotensinogen, *CACN1D* calcium channel voltage-dependent, L type, alpha 1D subunit, *CACNA1C* calcium channel voltage-dependent, L type, alpha 1C subunit, *CYP3A4* cytochrome P450 family 3 subfamily A member 4, *CYP3A5* cytochrome P450 family 3 subfamily A member 5, *GNB3* G Protein Subunit Beta 3, *NOS1AP* Nitric Oxide Synthase 1 Adaptor Protein, *NPPA* Natriuretic Peptide A, *NUMA1* nuclear mitotic apparatus protein 1, *PICALM* phosphatidylinositol binding clathrin assembly protein, *POR* cytochrome p450 oxireductase, *RYR3* Ryanodine Receptor 3, *TANC2* tetratricopeptide repeat, ankyrin repeat and coiled-coil containing 2

**Table 4:** Single nucleotide polymorphisms associated with BP response in genome-wide association analyses of hydrochlorothiazide-treated patients

Gene	SNP	Treatment outcome	Population	References
*ACE*	rs4303	BP response was altered by genotype.	African-American	[55]
*ADD1*	rs4961	BP response was altered by genotype carriers of the risk (T) allele. The T carriers responded better to low-dose diuretic therapy compared to the G allele risk carriers.	African-American, Caucasian and Asian	[55–64]
*WNK1* *NEDD4L*	rs880054 rs4149601 rs292499	SNP in *ADD1*, *WNK1*, and *NEDD4L*, when present in combination, may have significant effects on renal sodium handling, BP, and antihypertensive response to thiazides. However, variants in *WNK1* and *NEDD4*L genes have shown conflicting associations with BP.		
*AGT*	rs5051 rs699	T allele correlate with a higher incidence of uncontrolled hypertension and it is more frequent in African population compared to Caucasian.	African-American and Caucasian	[65]
*AGTR1*	rs5186	In African-American females, A allele is associated with increased reduction in BP.	African-American and Caucasian	[66]
*ALDH1A3*	rs3825926	SNP influenced response to hydrochlorothiazide treatment, 101445441A/G.	Caucasian	[67, 68]
*ANKFN1*	rs9915451	G allele has a decreased response to treatment in people with uncontrolled hypertension compare to the A allele.	Caucasian	[69]
*CLIC5*	rs321329	SNP influenced response to hydrochlorothiazide treatment, 45722988A/G.	Caucasian	[67, 70]
*CSMD1* and *CUB*	rs2776546 and rs11993031	AA genotype increased BP response to HCTZ compared to AT and TT genotype.	Caucasian	[69]
*DOT1L*	rs2269879	SNP was strongly associated with greater SBP and DBP blood in Caucasians.	Caucasian	[71]
*FBXL17*	rs4431329	Patients with the AA genotype and essential hypertension may have an increased response when treated with hydrochlorothiazide as compared to patients with the AT or TT genotypes.	Caucasian	[69]
*FGF5*	rs1458038	The T allele near *FGF5* was associated with higher BP and higher risk for HTN in Caucasian individuals. The authors found that the CC genotype better respond to anti-HTN drugs compared to the TT and TC genotype. This SNP was also strongly associated with BP in East Asians and South Asians and only marginally in African-Americans. Caucasian hypertensive individuals with the risk allele for HTN (T) might respond better to atenolol, than to HCTZ.	African-American, Caucasian, and Asian	[72–74]
*FTO*	rs4784333	C allele increased uric acid levels in subjects on HCTZ monotherapy plus subjects who started treatment on atenolol and then had HCTZ added. Result was not significant after Bonferroni correction.	African-American	[75]
*GNAS-EDN3*	rs2273359	Carriers of the CG genotype at this locus had a better BP response compared to the CC genotype.	African-American and Caucasian	[76]
*FOS* *DUSP1* *PPP1R15A*	rs11065987	Representative SNP of rs11065987, rs653178, rs10774625, and rs11066301, all of which are in high linkage disequilibrium with each other. The A allele of rs11065987 was associated with a greater decrease in both systolic and diastolic blood pressure following treatment with HCTZ compared to the GG genotype.	Caucasian	[77]
*GPR83*	rs3758785	The AA genotype may have increased response to hydrochlorothiazide in people with essential hypertension as compared to patients with genotype GG or AG.	African- American	[78]
*H3K4me1*	rs11750990	SNP identified to be associated with SBP response in the GWAS meta-analysis of Caucasians.	Caucasian	[55, 69, 72]
*HSD3B1*	rs6203 rs3765945 rs1047303	CC genotype associated with higher BP response and more significant in males. TC haplotype of SNP rs3765945 and rs1047303 have been significantly associated with SBP.	African-American	[72]
*KCNJ1*	rs59172778	SNP increase fasting blood glucose levels.	African-American and Caucasian	[79, 80]
*LUC7L2*	rs6947309	SNP elevated in African-Americans but not Caucasians.	African-American and Caucasian	[75]
*NEDD4L*	rs4149601	Carriers with the G allele have a greater BP reduction as compared to the AA homozygotes.	African-American and Caucasian	[81, 82]
*PRKCA*	rs16960228	BP responses were consistently greater in carriers of the A allele than in GG homozygotes.	Caucasians	[76, 83, 84]
*SH2B3*	rs3184504	The C allele (nonsynonymous, T/C, Trp/Arg) was associated with reduced BP in Caucasians, whereas in African-Americans the C allele is associated with a slight increase in blood.	African-American and Caucasian	[74]
*STK39*	rs6749447	The A minor allele has been associated with 2.0 mmHg higher SBP and a 1.0 mmHg higher DBP.	Caucasian	[85, 86]
*TET2*	rs12505746	SNP in GENRES cohort was associated with DBP, but not with SBP.	Caucasian	[69]
*TTC6*	rs177852	CC + CT are associated with increased response to HCTZ in people with HTN as compared to genotype TT. As greater antihypertensive efficacy of HCTZ was observed in Blacks compared to Whites.	African-American	[72]
*UGGT2*	rs9590353	SNP was associated with a lower decrease in DBP pressure after HCTZ treatment.	Caucasian	[69]
*12q Loci* *LYZ*, *YEATS4*, *FRS2*	rs317689, rs315135, rs7297610	Haplotype (ATC) was more frequently observed in the African population was more frequently observed in the African population, whereas the poor responders had either an ACT or ATT haplotype.	African-American and Caucasian	[81, 87, 88]

*ACE* angiotensin I converting enzyme, *ADD1* adducin 1, *AGT* angiotensinogen, *AGTR1* Angiotensin II Receptor Type 1, *ANKFN1* Ankyrin Repeat And Fibronectin Type III Domain Containing 1, *ALDH1A3* aldehyde dehydrogenase 1 family member A3, *ARDB1* adrenoceptor beta 1, *CDCA* Cell Division Cycle Associated Gene, *CLIC5* chloride intracellular channel 5, *CSMD1* CUB and Sushi multiple domains 1, *CSMD3* CUB and Sushi multiple domains 3, *CYP11B2* Cytochrome P450 Family 11 Subfamily B Member 2, *DIAPH3* Diaphanous Related Formin 3, *DOT1L* DOT1 Like Histone Lysine Methyltransferase, *EBF1* Early B Cell Factor 1, *ERCC6L2* ERCC Excision Repair 6 Like 2, *EDN3* endothelin 3, *FBXL17* F-Box And Leucine Rich Repeat Protein 17, *FGF5* fibroblast growth factor 5, *FOXA1* forkhead box A1 gene, *FTO* FTO, Alpha-Ketoglutarate Dependent Dioxygenase, *GJA1* gap junction protein α1 gene, *Gly460Trp* alpha-adducin, *GNAS* guanine nucleotide binding protein, alpha stimulating complex, *GPR83* G Protein-Coupled Receptor 83, *GRK5* G Protein-Coupled Receptor Kinase 5, *H3K27ac* Histone H3K27 acetylase, *H3K4me1* Histone H3K methylase, *HS3ST4* Heparan Sulfate-Glucosamine 3-Sulfotransferase 4, *HSD3B1* hydroxy-delta-5-steroid dehydrogenase, 3 β- and steroid δ-isomerase 1 gene, *HTN* hypertension, *KCNJ1* potassium voltage-gated channel subfamily J member 1, *LUC7L2* LUC7 Like 2, Pre-MRNA Splicing Factor, *NEDD4L* neural precursor cell expressed, developmentally down-regulated 4-like, E3 ubiquitin protein ligase, *NELFCD* negative elongation factor complex member C/D, *NOS3* Nitric Oxide Synthase 3, *PRKCA* protein kinase C alpha, *RAD52* RAD52 Homolog, DNA Repair Protein, *SBP* systolic BP response, *SH2B3* SH2B Adaptor Protein 3, *SLC12A3* solute carrier family 12 member 3, *SLC25A31* Solute Carrier Family 25 Member 31, *SNP* single nucleotide polymorphism, *STK39* serine/threonine kinase 39, *TCF7L2* Transcription Factor 7 Like 2, *TET2* Tet methylcytosine dioxygenase 2, *TTC6* Tetratricopeptide Repeat Domain 6, *UGGT2* UDP-Glucose Glycoprotein Glucosyltransferase 2, *YEATS4* YEATS domain containing 4

### Genetic association and pharmacogenomics of amlodipine response

The effect of calcium channel blockers in cardiac disease was first pioneered by Fleckenstein and colleagues (1983), using these drugs to reduce calcium influx into the heart in order to improve contractile function without impairing sodium dependent action potential [89]. As previously discussed, CCBs bind mainly to the L-type calcium cannels, which are located on the cardiac smooth muscle cells, causing inhibition of calcium ions into vascular smooth muscles leading to muscle relaxation and vasodilation [90]. This decrease in vascular resistance is known to reduce arterial BP with an associated decrease in HTN. However, SNPs within these genes are known to influence the efficacy of these drugs. In particular, the β2-regulatory subunit of the L-type calcium channel (*CCNB2*), β1-adrenergic receptor *(ADRB1*), and family of HECT domain E3 ubiquitin ligases (*NEDD4L*) have been identified, through GWAS and pharmacogenomics, as important genes in the regulation of BP in response to anti-HTN drugs [91]. However, to date none of these has been consistently replicated across studies [92].

Based on extracted data (Table 3), polymorphisms in genes coding for enzymes involved in the metabolic break down of amlodipine appear to play a major role in affecting its activity. For example, scientific evidence has suggested that cytochrome P450 family 3 subfamily A member 5 (*CYP3A5*) gene polymorphisms have significant association with anti-HTN response in patients treated with amlodipine [40, 46, 49]. Cytochrome (CYP) P450 iso-enzymes are drug-metabolizing enzymes that are known to play a pivotal role in the metabolic clearance of amlodipine. The subfamily of P450 iso-enzymes, *CYP3A4* and *CYP3A5* genes, is highly polymorphic and has been implicated in the observed variation in amlodipine response [93]. In addition, depending on the population under study, *CYP3A4/5* has been shown to display ethnic differences and gender differences that may affect the efficacy of amlodipine [42, 46, 47]. It has been reported that the *CYP3A4* genotype influences BP response of amlodipine in the African-American population, while *CYP3A5* SNPs have been linked to Korean and Chinese subjects [22, 46, 48, 49]. In the study conducted by Bhatnagar et al. (2010), it was shown that *CYP3A4* 392A/G promoter polymorphism was predictive of BP response among African-American women, where women with an A allele were three times more likely to reach a mean arterial pressure (MAP) of 107 mmHg [46]. This study suggests that the G allele carriers were rapid metabolizers of amlodipine. However, the results obtained from this study have been difficult to confirm in other independent populations and ethnic groups as the G allele at 392A/G has shown contradictory results pertaining to drug dose requirements. However, most studies did not examine the relationship in a gender-specific manner. The authors further found that the effects of *CYP3A4* 392A/G genotype on therapeutic response to amlodipine were not the same in men and women, suggesting differential CYP activity in the male versus female hormonal milieu. P-glycoprotein (PgP) levels, a multidrug transporter that mediates cellular transport of several drugs, have been shown to be two to three times higher in men in comparison to women. P-glycoprotein-mediated efflux of drugs such as amlodipine may result in higher intracellular concentrations in women, resulting in more variation in metabolism according to *CYP3A4* genotype. Furthermore, it has been reported that unlike *CYP3A4*, *CYP3A5* is highly polymorphic in Korean males (carriers) with a CYP3A5*3/*3 genotypes displaying lower plasma amlodipine concentration compared to the CYP3A5*1/*1 expressers, suggesting that *CYP3A5* polymorphisms have an effect on amlodipine disposition [48]. Nonetheless, conflicting data has been presented regarding observed individual variability to amlodipine and polymorphisms associated with *CYP3A4/5* genes. Though Kim et al. (2006) elegantly showed that CYP3A5*3 polymorphism affects efficacy of amlodipine, Guo and colleagues (2015) presented data showing that CYP3A5*3 polymorphism has no impact on plasma concentration and efficacy of amlodipine [42]. This was confirmed in an in vitro study done on human liver microsomes where the authors showed that *CYP3A4* is primarily responsible for amlodipine dehydrogenation, and that polymorphic expression of *CYP3A5* does not affect the pharmacokinetic variability of amlodipine [22]. Thus, to date no conclusive evidence is able to link the *CYP3A4/5* gene polymorphism across the different population groups to the observed variation in BP response and efficacy of amlodipine. Guo et al. (2015) also reported on genetic polymorphism in cytochrome p450 oxireductase (*POR*) and the effect of *POR* A-503V genetic polymorphism on CYP activity to influence the plasma concentration of amlodipine. However, due to the limitation of the sample size used in the study, the effect of the polymorphism could not be confirmed.

Amlodipine has also been shown to affect the drug transporter, ATP-binding cassette subfamily B member 1 (*ABCB1*) [40, 41, 94]. *The ABCB1* gene (also known as multi drug resistance 1; MDR1) is a known drug efflux pump with a role in the cellular accumulation of amlodipine. Not only do polymorphisms in this transporter affect amlodipine’s pharmacokinetics, but expression of this gene is also known to be affected by gender differences [40, 95]. In the latter study, the authors concluded that males with polymorphisms in the *ABCB1* genes require higher concentration of amlodipine compared to females. This study was on par with Kim and colleagues (2007), who showed that polymorphism in the *ABCB1* gene influences bioavailability and causes reduced plasma levels of amlodipine. Similarly, Zuo et al. (2014) observed that the *ABCB1* gene polymorphism C-3435T (rs1045642) might affect the plasma concentration of amlodipine in hypertensive patients in a Chinese Han population. However, as previously stated, the authors concluded that the polymorphisms did not have any impact on the efficacy of amlodipine. As limited studies have been conducted on the effect of the *ABCB1* transporter, no association can be made to the frequency of polymorphism in the anticipated gene and the effect thereof amlodipine efficacy.

Similar to *ABCB1*, other SNPs, such as those specific for the calcium channel, voltage-dependent, L type, alpha 1C subunit (*CACNA1C*), have been shown to influence the efficacy of amlodipine [45]. The authors investigated the efficacy of various CCB (including amlodipine) on 120 Caucasian subjects, diagnosed with HTN. Results obtained showed that three variance in *CACNA1C* had a significant response to amlodipine and felodipine, with rs2238032 and rs2239050 showing significant association with uncontrolled HTN. However, due to small sample size and homogeneous population, the authors concluded that results obtained, though significant, requires a more heterogeneous population to be confirmed. Furthermore, in a Japanese retrospective study cohort consisting of 48 randomly selected patients with controlled and uncontrolled HTN, the authors screened the promoter as well as all exons of the calcium channel, voltage-dependent, L type, alpha 1D subunit (*CACNA1D*) and *CACNA1C* genes. Three prominent SNP were identified (rs312481 and rs3774426 linked to *CACNA1D* and rs527974 linked to *CACNA1C*) conferring sensitivity to amlodipine and other CCB in patients with uncontrolled HTN [43, 44]. However, none of the identified SNPs were similar to those identified in the Brenner study (2006). Inferring that the SNPs identified in both studies were unique to their respective population, and thus a larger heterogeneous prospective study is needed to confirm the importance of these SNP in uncontrolled HTN.

Some of the SNPs summarized in this review for which greater information is still required to better inform of their effect on influencing the efficacy of amlodipine include phosphatidylinositol binding clathrin assembly (*PICALM*), tetratricopeptide repeat, akyrin repeat and coiled-coil containing 2 (*TANC2*), and nuclear mitotic apparatus protein 1 (*NUMA*). Briefly, in a GWAS performed on samples obtained from patients that participated in the HOMED-BP study [96], it was found that SNPs in three genes, *PICALM*, *TANC2*, and *NUMA*, were associated with BP response to amlodipine [44]. This study was replicated and the SNPs were confirmed in the multicenter GEANE study on amlodipine treatment [44]. However, this study must be interpreted within the context of its potential limitation in that only 265 Japanese patients were analyzed initially and this number decreased by more than half upon replication of the study. Additional SNPs identified are represented in Table 3.

### Genetic association and pharmacogenomics of HCTZ response

As previously mentioned, thiazide and thiazide-like diuretics are currently considered first-line medications for the treatment of HTN. However, like amlodipine, their therapeutic efficacy can be hindered by inter-individual variation. For instance, African-Americans and Caucasians with rs9943291 on 3-hydroxy-3-methylglutaryl-CoA synthase (HMGCS) have been shown to present with elevated blood glucose levels after treatment with both chlorthalidone and HCTZ [97, 98]. Although both these drugs are equally effective in managing HTN [98], HCTZ is the more commonly prescribed thiazide diuretic. Likewise, increasing studies, presented in Table 4, have focused on identifying genomic variants associated with HCTZ treatment. Although dosage-related side effects of HCTZ are acknowledged, various factors such as inter-individual differences, age, gender, and ethnicity play an important role in treatment outcome. For example, the α-adducin (alpha-adducin) Gly460Trp polymorphism within the angiotensin-converting enzyme (*ACE*) gene is linked to the development of HTN and can predict the degree of inter-individual response to HCTZ [55]. Pertaining to ethnic differences, African-Americans and Caucasians treated with 25 mg per day of HCTZ showed a significant decreased BP after correcting for both race and gender. However, in this study, African-American females displayed a greater decline in BP compared to the Caucasian females, inferring that racial difference plays a role in BP control [99].

In a meta-analysis involving hypertensive European Americans treated with HCTZ, it was demonstrated that genetic variations in protein kinase C Alpha (*PRKCA*) and negative elongation factor complex member C/D (*NELFCD*) were associated with DBP and SBP response, respectively [76, 83]. Also, in a study done to identify gene loci that may affect responsiveness of 228 male patients from European decent on monotherapy on four classes of anti-HTN drugs (HCTZ, amlodipine, bisoprolol, and losartan), > 80 SNPs were identified [67]. Interestingly, the authors reported that none of the identified SNP was common to more than a single drug class and that the study was unable to identify pharmacogenomics association of genome-wide significance, with the exception of three SNPs: aldehyde dehydrogenase 1 family member A3 (*ALDH1A3*), chloride intracellular channel 5 (*CLIC5*) and genes [67]. The identified SNPs in *ALDH1A3* (rs3825926) and *CLIC5* (rs31329) are known to influence HCTZ responsiveness, while variants in the Nephrin (*NPHS1*) (rs3814995) gene influence losartan treatment. Of interest, this study was performed in a European cohort, and the authors stated that SNPs in two other family members of the *ADH* gene (*ALDH1A2 and ALDH7*) were found to be associated with the presence of HTZ in African-Americans, while *ALDH2* has been reported to be associated with BP control in an East Asian population [67, 100, 101].

In recent years, GWAS studies conducted have identified particular loci associated with BP response to HCTZ within different ethnical groups [81, 87, 88]. In the African-American populations, a region of chromosome 12q15 was associated with HCTZ response. The haplotype analysis data suggested that SNPs associated with lysozyme (*LYZ*), Yeast Domain Containing 4 (*YEATS4*), and fibroblast growth receptor substrate 2 (*FRS2*) had an effect on HCTZ response in these patients [88]. This haplotype which was named ATC (a combination on alleles for SNPs rs317689 (A), rs315135 (T), and rs7297610(C)) was more frequently observed in the African-American population who showed a good response to HCTZ, whereas the poor responders had either an ACT or ATT haplotype [88]. The SNP rs7297610 (C/T) had the most significant association, and was considered to have the most noteworthy contribution to the observed phenotype. This association was replicated in a study of 291 African and 294 Caucasian individuals [81, 87]. In the PEAR study [81], the authors investigated the ATT haplotype and observed that the African-American population had a good HCTZ response; however, this was not observed in the Caucasian population. In addition, there was a reduction in the levels of *YEATS* expression in African-Americans after HCTZ treatment that were CC homozygotes for the SNP rs7297610, and not in the T carriers, which suggest a relationship between this Y*EATS* variant and HCTZ response [81, 87]. However, the study was not conclusive due to the sample size used in this investigation.

According to Salvi et al. (2017), the following studies were the largest collaborative studies conducted on the identification of novel loci that influence BP response to HCTZ. The major international study cohorts that contributed data to the meta-analysis included the following: the GENRES study [67]; the GERA-1 study [99], the HCTZ-Milan study [69]; the NORDIL study [82]; the PEAR-1; and the PHSS study [69]. The groups used a genome-wide meta-analysis approach, with validation in the African population, and they identified two candidate regulatory regions that were linked to the gap junction protein α1 gene (*GJA1*) and forkhead box A1 gene (*FOXA1*), two genes which were relevant to cardiovascular and kidney function. In addition, they implored a gene-based approach, from which they identified hydroxy-delta-5-steroid dehydrogenase, 3 β- steroid δ-isomerase 1 gene (*HSD3B1*) as being significantly associated with BP response [72]. Multiple studies have previously described the association of genetic variants in *HSD3B1* with HTN or BP variation [102–104]. The CC genotype at rs6203 was associated with HTN [105] and higher BP [102–104]. The latter association was reported as being more noteworthy in males and was confirmed by the authors’ single-GWAS studies [72], while SNP rs3765945 and rs1047303 were significantly associated with SBP [103, 104].

As previously stated, uncontrolled BP is a known risk for cardiovascular dysfunction. Svensson-Farbon (2017) reported on two genes demonstrating the strongest documented associations between BP lowering and adverse cardiovascular outcomes: neural precursor cell expressed, developmentally down-regulated 4-like, E3 ubiquitin protein ligase (*NEDD4L*) and *ADRB1* with β-blockers. In *NEDD4L*, the genetic polymorphism, rs4149601G/A, produces a cryptic splice site in *NEDD4L*, with the G allele resulting in down-regulation of the epithelial sodium channel (*ENaC*), thereby increasing sodium retention/reabsorption, a process that is important for regulation of chronic HTN [82]. The authors report that carriers of the G allele of this polymorphism (SNPs rs4149601 and rs292499) were associated with a greater BP lowering response to HCTZ when compared to the AA homozygotes (Svensson-Farbom et al., 2011). In addition, increased risks for adverse cardiovascular outcomes were identified in Caucasians that were AG heterozygous for rs4149601, and not treated with HCTZ. The hypothesis, that patients with the rs4149601G/A polymorphism will exhibit a greater response to thiazides, has been confirmed by an independent study where the carries of G allele carriers were compared to that of the AA genotype in a Caucasian population in the PEAR-1 study [81]. However, further research is needed to fully understand whether this polymorphism could be used to guide treatment decisions in patients with HTN, as the available data are certainly compelling.

For the gene *PRKCA*, the combined meta-analysis of data obtained from the PEAR-1, GERA-1, NORDIL, and GENRES studies, all displayed genome-wide significance for rs16960228 [67, 76, 87]. Individuals who were carriers of the A allele showed greater BP response to HCTZ then the GG homozygote and had an increased level of *PKRCA* expression in Caucasians. The authors then continued and investigated the contribution of other *PRKCA* SNPs to BP response and found an additional SNP within the *PRKCA* gene to be associated with BP control in African-American. Pertaining to the PEAR study, three BP lowering alleles associated with BP response to HCTZ monotherapy were identified [74]. The three SNPs that were identified in the genes include fibroblast growth factor 5 (*FGF5*—rs1458038), SH2B Adaptor Protein 3 gene (*SH2B3*—rs3184504), and Early B Cell Factor (*EBF1*—rs45551053) in a Caucasian population. The allele of rs1458038 near *FGF5* was associated with higher BP and increased risk for HTN in Caucasian individuals [74]. The authors found that the CC genotype responded better to anti-HTN drugs compared to the TT and TC genotype. Gong (2012) also reported that Caucasian hypertensive individuals with the T risk allele for HTN might respond better to atenolol, than to HCTZ. This SNP was also strongly associated with BP in East Asians and only marginally in African-Americans [73]. Also, Gong (2012) reported that the C allele (nonsynonymous, T/C, Trp/Arg) in *SH2B3* was associated with reduced BP in Caucasians, but in African-Americans the C allele is associated with a slight increase in blood [74].

In a 2013 GWAS study done by Turner et al. (2013), it was shown that the *GNAS–EDN3* region variant rs2273359 was significantly associated with SBP response to HCTZ in the PEAR, GERA, and NORDIL studies [76]. In the three studies, the available data indicated that carriers of the CG genotype at this locus had an improved BP response to HCTZ than those with the CC genotype. Additionally in a recent study performed by Sa and colleagues (2018), the authors identified three genes, FBJ murine osteosarcoma viral oncogene homolog (*FOS*), dual specificity phosphatase 1 (*DUSP1*), and protein phosphatase 1 regulatory subunit 15A (*PPP1R15A*), as potential molecular determinants of antihypertensive response to thiazide diuretics [77]. The authors found that the representative SNP of rs11065987, rs653178, rs10774625, and rs11066301 were all in high linkage disequilibrium with each other. An allele of rs11065987 was associated with a greater decrease in both SBP and DBP compared to the GG genotype following treatment with HCTZ. In a GWAS performed in Amish individuals, the authors found a strong association with common variants in a serine/threonine kinase 39 (STK39) showing that variants in STK39 may influence BP by increasing STK39 expression and consequently altering renal Na^+^ excretion, thus unifying rare and common BP-regulating alleles in the same physiological pathway [86].

However, results obtained in most of these studies are inconsistent and mostly conflicting due to issues relating to population stratification, study design and sample size, exposure to antihypertensive drugs, and the different methodologies used in the respective studies [78, 106, 107]. It is a well-accepted fact that most of the cardiovascular-associated phenotypes, including HTN, are polygenic in nature as have been demonstrated in individuals of European descent in a multistage GWAS study that identified 29 previously known or new SNP variants that influence BP [73]. This polygenic nature is one of the biggest obstacles in identifying the most appropriate SNPs associated with uncontrolled hypertensions between different ethnic groups.

Similarly, in a study performed by Manunta (2008), the authors identified three variants in genes that play a role in the regulation of renal sodium absorption: α-adducin *ADD1* (rs4961), *WNK1* (rs880054 A/G), and *NEDD4L* (rs4149601 G/A). The identified genes have been associated with increased BP; however, findings have been inconsistent. However, the authors found that when individuals carry specific alleles of the three genes in combination, a marked response to thiazide treatment could be observed [58–64].

Furthermore, additional SNP in candidate genes listed in Table 4 has been shown to influence HCTZ response in hypertensive patients and include alpha-adducin (*ADD1*; T allele carriers responded better to low-dose diuretic therapy) [56], rs5051 SNP in the promoter of the angiotensin gene (*AGT*) and the linkage with rs699, which results in a M235T variant, also referred to as C4072T, has been associated with an increased risk of hypertension in African-Americans compared to Caucasians. However, the C allele in rs699 has been reported to be associated with an increased risk of pregnancy-induced HTN (pre-eclampsia) in Caucasians [57, 65]. Ankyrin-repeat and fibronectin type III domain containing 1 (*ANKFN1*; G allele carriers have a decreased response to HCTZ in Caucasians), Angiotensin II type 1 receptor (*AGTR1*, A allele has an increased response to HCTZ treatment in both African-Americans and Caucasians), UDP-glucose glycoprotein glucosyltransferase 2 (*UGGT2*; SNP rs9590353 was associated with a lower decrease in DBP after HCTZ treatment), Calmodulin Ubiquitin (CUB) and Sushi multiple domains 1 (*CSMD1*, T allele has a decreased response to HCTZ compared to A allele in Caucasians), Tet methylcytosine dioxygenase 2 (*TET2*, A allele has a decreased response to HCTZ compared to T allele in Caucasians), F-box and leucine rich repeat protein (*FBXL17*, T allele has a decrease response to HCTZ in Caucasians) [69]. SNPs associated with Luc7-like 2 (*LUC7L2*, SNP rs6947309 was associated with high BP in African-Americans) Potassium inwardly-rectifying channel, subfamily J, member 1 (*KCNJ1*, AG genotype associated with an increased response to HCTZ compared to AA genotype in both African-Americans and Caucasians) [79], tetratricopeptide repeat domain 6 (*TTC6,* C allele has an increased response to HCTZ compared to T allele), and alpha-ketoglutarate-dependent dioxygenase gene (*FTO*, C allele carriers of rs4784333 following HCTZ treatment showed an increased risk for hyperuriceamia in African-Americans) has also been shown to affect HCTZ treatment outcome [75].

Although alterations to most of these genes have been shown to have a significant effect on the BP lowering effect in response to HCTZ treatment, substantial evidence is still required to elaborate on the effect on inter-individual variation as very few of the SNPs identified have been replicated in more than one study and/or more than one population group. Therefore, further replications in other populations and functional studies are warranted to improve our understating of the patients genetic profile and its effect on drug treatment outcome.

## Conclusion

The current data on genetic polymorphisms in HTN genetics clearly indicates that no single genetic polymorphism with very large effect size exists and it is therefore recommended that two or more genes should be used to guide treatment outcomes, however to date no such genes exist. It is further recommended that a combination of modest effect size variants will probably need to be defined if we are to reach the goal of genetic-guided antihypertensive therapy. However, although our understanding of single gene effects on drug metabolism is progressing, our comprehension of multiple gene effects is very limited, and this complicates their use in diagnostics and improved treatment outcome. Therefore, despite predictions that we are entering the realm of personalized polymorphism-orientated drug therapy for most diseases in the near future, HTN and other complex disorders will require more extensive research before we reach that point. Furthermore, numerous additional reports frequently associate new SNPs with BP control, and some are removed as new scientific information becomes available.

For examples, the most recent release of dbSNP (database build 138) contains more than 38 million SNPs that span the human genome (https://www.ncbi.nlm.nih.gov/projects/SNP—accessed 20 September 2018). This creates an enormous challenge to investigate the potential effect of every SNP, despite the need to prioritize SNPs on the basis of variables that are informative. One approach is so use a tag-SNP approach (representative SNPs that are in high linkage disequilibrium with other variants) as adopted by the International HapMap Project and other GWAS studies [108, 109]. However, tag SNPs that are found to correlate with differences in drug response is not always the causative SNPs that are important for function of the drug-response gene. Due to this fact that they could serve no functional insight as pharmacogenetics prediction markers, one would therefore still have to identify the potentially functional SNPs in linkage disequilibrium with the tag SNPs. In addition to this, these SNPs will still need to be validated experimentally, and that is another major challenge to consider despite the increase in SNP functional prediction algorithms.

In addition, to date the development of a SNP-chip for HTN is still far off as literature curation of drug-response genes requires an extensive collaborative effort and may be subjected to a high error rate. The International Consortium for Antihypertensive Pharmacogenomics Studies (ICAPS) was created in 2012 to promote the collaboration among different research groups. The Consortium might facilitate the identification and replication of pharmacogenetic findings for the variability on antihypertensive drug response and might reveal strong signals to be further used to guide treatment selection (http://www.pharmgkb.org/page/icaps). Together, these efforts may reveal new therapeutic strategies and drug/dose optimization for HTN treatment. Furthermore, the pre-emptive availability of genetic information in patient’s medical records may favor the prescription based on genomic information that might be more cost-effective than the trial and error in current practice. The pharmacogenomics of antihypertensive drugs is a field in progress and future efforts are needed to unravel additional genes and variants, as well as identify epigenetic and regulatory pathways involved in the responsiveness to antihypertensive drugs. The potential of pharmacogenomics to improve treatment of all disease, and in particular uncontrolled HTN is enormous. The advantage of screening an individual who have undesired side effects could lead to an optimized treatment regimen that should assist in the improvement of patient outcome and adherence to treatment. However, the application of SNP genotyping and haplotyping will not necessarily eradicate all problems associated with treatment regiments, but could reduce the waste associated with wrong treatment. As such, these applications will also be useful in creating a pipeline for the treatment of patients as new drugs are discovered and developed.
